# CHI3L1 monoclonal antibody therapy mitigates cognitive impairment by inhibiting neuroinflammation through ERK and NF-κB pathway in Tg2576 mice

**DOI:** 10.3389/fnmol.2025.1728279

**Published:** 2026-01-07

**Authors:** Hyeon Joo Ham, Seung Sik Park, Yong Sun Lee, Tae Hun Kim, Dong Ju Son, Ji-Hun Kim, Key-Hwan Lim, Hanseul Park, Hye Jin Lee, Jaesuk Yun, Sang-Bae Han, Min Ki Choi, Jin Tae Hong

**Affiliations:** College of Pharmacy and Medical Research Center, Chungbuk National University, Cheongju, North Chungcheong, Republic of Korea

**Keywords:** Alzheimer’s disease, antibody therapy, CHI3L1, HAX1, neuroinflammation, NF-κB

## Abstract

**Introduction:**

Alzheimer’s disease (AD) is neurodegenerative disorder characterized by chronic inflammation in the brain. Chitinase-3-like 1 (CHI3L1), a secreted glycoprotein that is upregulated in a variety of diseases with chronic inflammation, represents a promising target for AD. Here, we studied the inhibitory effect of a novel CHI3L1 monoclonal antibody (H1) on memory impairment and neuroinflammation in Tg2576 transgenic mice.

**Methods and results:**

H1 was shown to cross the blood–brain barrier selectively, as confirmed by fluorescence imaging. Tg2576 mice were administered H1 (2 mg/kg, i.v., weekly for 1 month), and cognitive functions were assessed through behavioral tests. H1 treatment alleviated memory impairment and reduced amyloid deposition and neuroinflammation both in Tg2576 mice and Aβ-induced BV-2 microglial cells. Mechanistically, H1 inhibited the ERK and NF-κB signaling pathways and suppressed M1 microglial marker expression. Global proteomic analysis and gene expression profiling in BV-2 cells and Tg2576 mouse brains revealed a strong association between CHI3L1 and HAX1 expression. H1 therapy significantly reduced HAX1 levels in both *in vivo* and *in vitro* models. Moreover, HAX1 induction by Aβ or CHI3L1 was blocked by an NF-κB inhibitor.

**Discussion:**

These findings suggest that CHI3L1 monoclonal antibody therapy may attenuate cognitive decline in AD by modulating neuroinflamma.

## 1 Introduction

Alzheimer’s disease (AD) is the most common neurodegenerative disease, causing progressive impairment of cognitive function, impaired memory, and changes in behavior and emotional state (Cummings et al., 2018; Mangold and Szpara, 2019; Miao et al., 2023). Accumulation of Aβ and tau proteins in the brain is a representative pathological feature of AD and is considered one of the major factors in the pathogenesis of AD (Gulisano et al., 2018; Liu et al., 2019; Webers et al., 2019). Alongside the abnormal accumulation of Aβ and tau proteins, neuroinflammation is another major feature observed in the brains of AD patient (Heppner et al., 2015; Harach et al., 2017). This inflammatory response begins in the earliest stages of AD and has both beneficial and detrimental effects as the disease progresses (Heppner et al., 2015). The brain’s immune system perceives the abnormal protein buildup as a harmful stimulus, triggering the activation of immune cells and the release of inflammatory molecules (Heppner et al., 2015; Leng and Edison, 2021). Microglia play various roles in phagocytosis, inflammation, and neurodegeneration during the progression of AD (Miao et al., 2023). Aβ plaques bind to TLR2 or TLR4 on microglia, activating microglia to produce inflammatory cytokines, causing an inflammatory response (Trudler et al., 2010; Neniskyte et al., 2016). This inflammatory response contributes to Aβ plaque phagocytosis, but if this state persists and becomes chronic inflammation, it has been shown to worsen neurodegeneration through the continuous release of toxic substances and contribute to the progression of AD (Leng and Edison, 2021; Miao et al., 2023). Microglia can polarize into either the M1 pro-inflammatory phenotype or the M2 anti-inflammatory phenotype, referred to as classical activation and alternative activation, respectively (Wan et al., 2022). While M1 microglia release inflammatory cytokines and chemokines, leading to inflammation and neuronal death, M2 microglia are involved in tissue maintenance and repair, promoting anti-inflammatory and neuroprotective effects (Guo et al., 2022). Some studies reported that neuronal damage from excessive M1 microglia, the pro-inflammatory microglia, contributes to AD pathology (Yao and Zu, 2019).

Chitinase-3-like 1 (CHI3L1) is a chitinase-like protein with no enzymatic activity that belongs to the glycoside hydrolase 18 family, known as breast regression protein 39 (BRP-39) in mice and YKL-40 in humans (Connolly et al., 2023; Qu et al., 2024). CHI3L1 is produced by various cell types, such as macrophages, neutrophils, chondrocytes, synoviocytes, osteoblasts, and smooth muscle cells, along with central nervous system-specific cells, including neurons, astrocytes, and microglia (Qu et al., 2024; Mwale et al., 2025). It has been reported that the expression of CHI3L1 is induced by inflammatory stimuli and has been linked to disease involving chronic inflammation (Coffman, 2008; Querol-Vilaseca et al., 2017; Talaat et al., 2023). Multiple clinical studies have demonstrated that CHI3L1 levels are elevated in individuals with inflammatory conditions like Crohn’s disease, rheumatoid arthritis, osteoarthritis, and asthma, as well as in those with neurodegenerative disorders, including AD, Parkinson’s disease, and amyotrophic lateral sclerosis (Bonneh-Barkay et al., 2010; Yu et al., 2024). The levels of CHI3L1 in the cerebrospinal fluid (CSF) and plasma of AD patients were significantly higher than those in healthy controls, and its concentration increased as the disease progressed (Batinic et al., 2012; Muszynski et al., 2017; Hampel et al., 2018). Notably, CHI3L1 levels in CSF showed a stronger correlation in Aβ-positive patients, and higher CSF CHI3L1 levels were reported to be associated with an increased risk of developing AD dementia in individuals without dementia (Hampel et al., 2018; Janelidze et al., 2018). In the brain, CHI3L1 is primarily produced by activated microglia and astrocytes, reflecting inflammatory processes (Wennstrom et al., 2015; Jiang et al., 2023). CHI3L1 secreted by astrocytes in response to microglial activation functions as an inflammatory signaling molecule, mediating immune responses, and studies suggest that this signaling activity may contribute to neurodegeneration (Connolly et al., 2023). These findings indicate that CHI3L1 is involved in neuroinflammation, a key pathological feature of AD, and that its levels are elevated in AD patients compared to healthy individuals. However, the precise role of CHI3L1 in neuroinflammation and AD pathogenesis remains unclear.

HCLS1-associated protein X-1 (HAX1) is multifunctional protein expressed in most tissues participating in various physiological processes (Klein, 2017; Lu et al., 2018). HAX1 is known to be mainly involved in apoptosis, but whether the role of HAX1 is a pro-apoptotic regulator or an anti-apoptotic regulator is still controversial (Lu et al., 2018). Using the LC–MS/MS analysis for global proteomics, we found that HAX1 was associated with CHI3L1 in BV-2 cells. However, the role of HAX1 in AD and the involvement of HAX1 in the CHI3L1-mediated neuroinflammation still unclear.

Our previous study, we found that the CHI3L1 inhibitor alleviated memory dysfunction and neuroinflammation in Αβ-induced AD mouse model (Choi et al., 2018). However, CHI3L1 mAb therapy has not yet been studied. In the present study, we investigated the therapeutic effect of anti-CHI3L1 monoclonal antibody on Tg2576 AD mouse model and its mechanisms of action in terms of amyloidogenesis and neuroinflammation.

## 2 Materials and methods

### 2.1 Materials

The novel monoclonal antibody targeting CHI3L1 (CHI3L1 mAb, H1) was synthesized by New Drug Development Center of Osong Medical Innovation Foundation (Chungcheongbuk-do, Korea). The H1 was dissolved in PBS (final concentration of 3.9 mg/mL) and stored at −20 °C until use. The Aβ_1-42_ was purchased from Sigma Aldrich (St. Louis, MO, USA). The U0126, SP600125, SB203580, and Bay 11–7,082 were purchased from Sigma Aldrich (St. Louis, MO, USA).

### 2.2 Animal and treatment

Twelve-month-old Tg2576 mice were maintained and handled in accordance with the guidelines for animal experiments of the institutional animal care and use committee of the Laboratory Animal Research Center at Chungbuk National University, Korea (ethics approval No. CBNUA-1420-20-01). All efforts were made to minimize animal suffering and to reduce the number of animals used. All mice were housed in 4-mouse cages with automatic temperature control (21 °C-25 °C) at relative humidity levels of 45 to 65% with a 12-h light–dark cycle. Food and water were provided ad libitum. Tg2576 mice harboring human APP695 with Swedish double mutation (hAPP; HuAPP695; K670N/M671L) were purchased from Taconic Farms (Germantown, NY, USA), and the strain was maintained in the animal laboratory at Chungbuk National University. Tg2576 mice were randomly divided into two groups: (I) the control vehicle-treated group (*n* = 10, ♂3, ♀7) and (II) the H1 (2 mg/kg)-treated group (*n* = 10, ♂2, ♀8). In our previous study using the Tg2576 mouse model (Ham et al., 2019; Ham et al., 2020b), behavioral and inflammatory outcome measures achieved statistical significance with *n* = 10–13 animals per group. Consistent with these data, we selected a sample size of *n* = 10 mice per group for the present study. The H1 was administered through intravenous injection once a week for a month. Control mice were alternatively injected an equal volume of vehicle. The behavioral tests of learning and memory capacity were assessed using the water maze, probe, and passive avoidance tests. Mice were sacrificed after behavioral tests by CO_2_ asphyxiation.

### 2.3 *Ex vivo* imaging of ICG-labeled H1 antibody

The H1 was conjugated to ICG (Indocyanine green) using the ICG labeling kit (Dojindo, Kumamoto, Japan; Cat No. LK31), according to the manufacturer’s specifications. Freshly prepared ICG-labeled H1 (1 mg/kg) was injected into WT mice, WT mouse with LPS injection, and Tg2576 mouse via the tail vein. 15 min after administration, brains were isolated and washed with PBS. Fluorescence images were obtained using the VISQUE In Vivo Optical Imager system (VIEWORKS, Gyeonggi, Korea). The NIR filter set, which ranges between 740 to 790 nm (excitation) and between 810 nm to 860 nm (emission) were used for ICG fluorescence.

### 2.4 Morris water maze

The water maze test is a commonly accepted method for assessing cognitive function, and we performed as described by Morris (1984). Maze testing was carried out by the SMART-CS (Panlab, Barcelona, Spain) program and equipment. A circular plastic pool (height: 35 cm, diameter: 100 cm) was filled with water made opaque with skim milk kept at 22–25 °C. An escape platform (height: 14.5 cm, diameter: 4.5 cm) was submerged 1–1.5 cm below the surface of the water in position. Testing trials were performed on a single platform and at two rotational starting positions. Each trial lasted for 60 s or ended as soon as the mouse reached the submerged platform. After testing trial, the mice were allowed to remain on the platform for 120 s and were then returned to their cage. Escape latency and escape distance of each mouse were monitored by a camera above the center of the pool connected to a SMART-LD program (Panlab, Barcelona, Spain). A quiet environment, consistent lighting, constant water temperature and a fixed spatial frame were maintained throughout the experimental period.

### 2.5 Probe test

To assess memory retention, a probe test was performed 24 h after the water maze test. The platform was removed from the pool which was used in the water maze test, and the mice were allowed to swim freely. The swimming pattern of each mouse was monitored and recorded for 60 s using the SMART-LD program (Panlab, Barcelona, Spain). Retained spatial memory was estimated by the time spent in the target quadrant area.

### 2.6 Passive avoidance performance test

The passive avoidance test is generally accepted as a simple method for testing memory. The passive avoidance response was determined using a “step-through” apparatus (Med Associates Inc., Vermont, USA) that is divided into an illuminated compartment and a dark compartment (each 20.3 cm × 15.9 cm × 21.3 cm) adjoining each other through a small gate with a grid floor, 3.175 mm stainless steel rods set 8 mm apart. A training trial was performed 2 days after the probe test. For the training trial the mice were placed in the illuminated compartment facing away from the dark compartment. When the mice moved completely into the dark compartment, it received an electric shock (0.45 mA, 3 s duration). Then the mice were returned to their cage. One day after training trial, the mice were placed in the illuminated compartment and the latency to enter the dark compartment defined as “retention” was measured. The time taken for the mice entered into the dark compartment was recorded and described as step-through latency. The cut-off time limit of the retention trials was set at 3 min.

### 2.7 Collection and preservation of brain tissues

After the completion of all the behavioral tests, the mice were perfused with PBS with heparin under inhaled CO_2_ anesthetization. The brain was immediately removed from the skull of mouse, separated into left and right brain, and randomly allocated either for storage at −80 °C or fixation in a 10% formalin solution for 3 days at room temperature.

### 2.8 Thioflavin S staining

The brain fixed in a 10% formalin solution was embedded in paraffin wax, and then the brain was cut into sections 5-μm-thick slices. Thioflavin S staining was performed as described previously (Ham et al., 2019). The sections were mounted in a mounting medium (Vectashield^®^ mounting medium for fluorescence with DAPI; Vector laboratories, Burlingame, CA, USA). The thioflavin S staining was examined using a confocal fluorescence microscope (K1-Fluo; Nanoscope systems, Daejeon, Korea) (× 50 and × 200).

### 2.9 ELISA assay

Aβ_1-42_ and Aβ_1-40_ levels were determined using each specific mouse Aβ_1-42_ enzyme-linked immunosorbent assay (ELISA) Kit and mouse Aβ_1-40_ ELISA Kit purchased from CUSABIO (Houston, TX, USA; Cat No. CSB-E10787m and CSB-E08300m, respectively) following the manufacturer’s protocol.

### 2.10 Assay of β-secretase activities

β-secretase activity in the mice brains was determined using a commercially available β-secretase activity kit (Abcam, Inc., Cambridge, MA, USA; Cat No. ab65357). Solubilized membranes were extracted from hippocampus tissues using β-secretase extraction buffer, incubated on ice for 1 h and centrifuged at 5000 × g for 10 min at 4 °C. The supernatant was collected. A total of 50 μL of sample (total protein 100 μg) or blank (β-secretase extraction buffer 50 μL) was added to each well (used 96-well plate) followed by 50 μL of 2X reaction buffer and 2 μL of β-secretase substrate incubated in the dark at 37 °C for 1 h. Fluorescence was read at excitation and emission wavelengths of 335 and 495 nm, respectively, using a fluorescence spectrometer (Gemini EM; Molecular Devices, CA, USA).

### 2.11 Western blot analysis

Homogenized brain hippocampus tissues were lysed by protein extraction solution (PRO-PREP, iNtRON, Kyungki-do, Korea) and the total protein concentration was determined using the Bradford reagent (Bio-Rad, Hercules, CA, USA). 40 μg of extracted protein were separated by SDS/PAGE and transferred to Immobilon^Ⓡ^ PVDF membranes (Millipore, Bedford, MA, USA). The membrane was blocked with 5% BSA in Tris-buffered saline containing 0.05% Tween-20 (TBST) for 1 h at room temperature, followed by incubation with specific primary antibodies for overnight at 4 °C. The membranes were washed with TBST and incubated with diluted HRP-conjugated secondary antibodies for 1 h at room temperature. After washes, binding of antibodies to the PVDF membrane was detected using the Immobilon Western Chemiluminescent HRP Substrate (Millipore, Bedford, MA, USA). The band intensities were measured using the Fusion FX 7 image acquisition system (Vilber Lourmat, Eberhardzell, Germany) and quantified using Image J software. To detect target proteins, specific primary antibodies against iNOS, IBA-1, GFAP, APP, and BACE1 (1:1000; Abcam, Inc., Cambridge, UK; Cat No. ab3523, ab178846, ab7260, ab32136, and ab183612, respectively), COX-2 and HAX1 (1:1000; Novus Biologicals, Inc., CO, USA; Cat No. AF4198 and NBP2-57945, respectively), ERK 1/2, p-ERK 1/2, JNK, p38, p-p38, IκBα, p-IκBα, and Myc-tag (1:1000; Cell signaling Technology, Inc., MA, USA; Cat No. 4695, 4,370, 67,096, 8,690, 4,511, 4,814, 5,209, and 2,276, respectively), p-JNK and β-actin (1:200; Santa Cruz Biotechnology Inc., Santa Cruz, CA, USA; Cat No. sc-6254 and sc-47778, respectively) were used. The corresponding conjugated secondary antibodies such as anti-mouse, anti-rabbit and anti-goat purchased from Abcam (Cambridge, UK; Cat No. ab6789, ab6721, and ab150129, respectively).

### 2.12 Immunohistochemistry

The brain fixed in a 10% formalin solution was embedded in paraffin wax, and then the brain was cut into sections 5-μm-thick slices. Immunohistochemistry was performed as described previously (Ham et al., 2019). To detect target proteins, specific antibodies against GFAP, IBA-1, iNOS (1:250; Abcam, Inc., Cambridge, MA, USA; Cat No. ab7260, ab178846, and ab3523, respectively), and COX-2 (1:100, Novus Biologicals, Inc., CO, USA; Cat No. AF4198) were used. Brain sections were visualized by a chromogen diaminobenzidine (Vector Laboratories, Burlingame, CA, USA). Finally, brain sections were mounted with mounting medium Cytoseal XYL (Thermo Scientific, Waltham, MA, USA), and evaluated on a light microscope (Microscope Axio Imager. A2; Carl Zeiss, Oberkochen, Germany; × 50 and × 200).

### 2.13 Quantitative real-time PCR

The mRNA level was measured by quantitative real-time polymerase chain reaction (qRT-PCR). Total RNA was extracted using RiboEX (Geneall biotechnology, Seoul, Korea) from hippocampus tissue and cDNA was synthesized using High-Capacity cDNA Reverse Transcription kit (Thermo Scientific, Waltham, MA, USA). Quantitative real-time PCR was performed on a 7,500 real-time PCR system (Applied Biosystems, Foster City, CA, USA) for custom-designed primers and β-actin was used for house-keeping control using HiPi Real-Time PCR SYBR green master mix (ELPIS biotech, Daejeon, Korea). Cycling conditions consisted of a initial denaturation step of 3 min at 94 °C, a denaturation step of 30 s at 94 °C, an annealing step of 30 s at 56 °C and an extension step of a minute at 72 °C followed by 40 cycles. The values obtained for the target gene expression were normalized to β-actin and quantified relative to the expression in control samples. Each sample was run with the following primer pairs shown in Supplementary Table S1.

### 2.14 BV-2 microglial cell culture

Microglial BV-2 cells were obtained from the American Type Culture Collection (Rockville, Maryland, United States). Microglial BV-2 cells were maintained with serum-supplemented culture media of DMEM supplemented with FBS (10%) and antibiotics (100 units/mL). The microglial BV-2 were incubated in the culture medium in a humidified incubator at 37 °C and 5% CO_2_. The cultured cells were treated with several concentrations (10, 20, 50, and 100 ng/mL) of H1, 2 h before Aβ (5 μM) treatment. The cells were harvested after 24 h.

### 2.15 Transfection

BV-2 cells were transiently transfected with siRNA (20 nM/well/6-well plate) or using the Lipofectamine^®^ RNAiMAX transfection reagent in Opti-MEM, according to the manufacturer’s specification (Invitrogen, Waltham, MA, USA). BV-2 cells were transiently transfected with pcDNA3.1(+)-6 × Myc-CHI3L1 vector (1 μg/well/6-well plate) or control vector using the Lipofectamine^®^ 3,000 transfection reagent in OPTI-MEM, according to the manufacturer’s specification (Invitrogen, Waltham, MA, USA). Negative control (NC), CHI3L1 siRNA were purchased from OriGene Technologies, Inc. (Rockville, MD, USA). pcDNA3.1(+)-6 × Myc-CHI3L1 vector was cloned from Bionics (Seoul, Republic of Korea).

### 2.16 LC–MS/MS for global proteomics

BV-2 cells were transiently transfected with pcDNA3.1(+)-6 × Myc-CHI3L1 vector (1 μg/well/6-well plate) or control vector. After 24 h, the cells were harvested and lysed by 8 M Urea (Merck, Branchburg, NJ, USA) in room temperature. The total protein concentration was determined using the Bradford reagent (Bio-Rad, Hercules, CA, USA). 100 μg of extracted protein in 100 μL of 8 M Urea were reduced with 10 mM dithiothreitol (Sigma Aldrich, St. Louis, MO, USA) and alkylated 25 mM iodoacetamide (Sigma Aldrich, St. Louis, MO, USA) for 1 h at each step. Samples were diluted by adding 900 μL of 1 M Tris (pH 8.0) and trypsinized (Promega, Madison, WI, USA) for 17 h at 37 °C (trypsin/protein ratio of 1:50, w/w). The resulting peptide mixture was lyophilized overnight and digested peptides were cleaned by flowing through a Oasis HLB 1 cc (10 mg) solid phase extraction (SPE) cartridges (Oasis, Milford, MA, USA). Samples were dried using a Speed-Vac (Thermo Savant, Holbrook, NY) overnight. The samples were resuspended by adding 200 μL of 0.1% formic acid (Sigma Aldrich, St. Louis, MO, USA) and centrifuged at 14,000 rpm for 5 min at 4 °C. The supernatant was used for LC–MS/MS analysis. Nano-high-performance liquid chromatography (nano-LC) analysis were performed using an Easy n-LC 1000 system (Thermo Scientific, Waltham, MA, USA). The PepMap RSLC column (15 cm x 75 μm) have 2 μm, 100 Å particle size C_18_ beads (Thermo Scientific, Waltham, MA, USA). The mobile phase consisted of water containing 0.1% formic acid (A) and acetonitrile containing 0.1% formic acid (B). The flow rate was 0.3 μL/min. The gradient elution profile was as follows: 0.0–10.0 min (A: 95%, B: 5%); 10.0–45.0 min (A: 95–70%, B: 5–30%); 45.0–57.0 min (A: 70–10%, B: 30–90%); and 57.0–70.0 min (A: 10–95%, B: 90–5%). An Q-ExactiveTM mass spectrometer (Thermo Scientific, Waltham, MA, USA) was used for MS analyses and was operated with Xcalibur (version 2.1) to generate peak lists. For peptide ionization, 2,400 V was applied and a 250 °C capillary temperature was used. The full scan event was collected using a m/z 350–2000 mass selection, an Q-Exactive MS resolution of 70,000, a target automatic gain control (AGC) value of 1 × 106, and a maximum injection time of 80 ms. Fragmentation was performed with a normalized collision energy of 25. Proteins were functionally classified using a gene ontology system by biological processes, molecular activities and cellular components using the protein discoverer 2.2 program (Thermo Scientific, Waltham, MA, USA) and normalized protein abundance values were calculated.

### 2.17 Statistical analysis

The data were statistically analyzed using the GraphPad Prism software (Version 4.03; GraphPad software, Inc., San Diego, CA, USA). Data are presented as mean ± S. E. M. The group differences in all data were assessed by Student’s t test or one-way analysis of variance (ANOVA) followed by the Tukey multiple comparison test. A value of *p* < 0.05 was considered statistically significant. *, Significantly different between two groups (*p* < 0.05). ** Significantly different between two groups (*p* < 0.01). *** Significantly different between two groups (*p* < 0.001).

## 3 Results

### 3.1 CHI3L1 mAb attenuated memory dysfunction in Tg2576 mice

*Ex vivo* imaging was performed to determine if the H1 antibody could be localized to the brain. We found that H1 was able to penetrate brain tissue only in the Tg2576 AD mouse model and LPS induced AD-like mouse model (Supplementary Figure S1).

**Figure 1:**
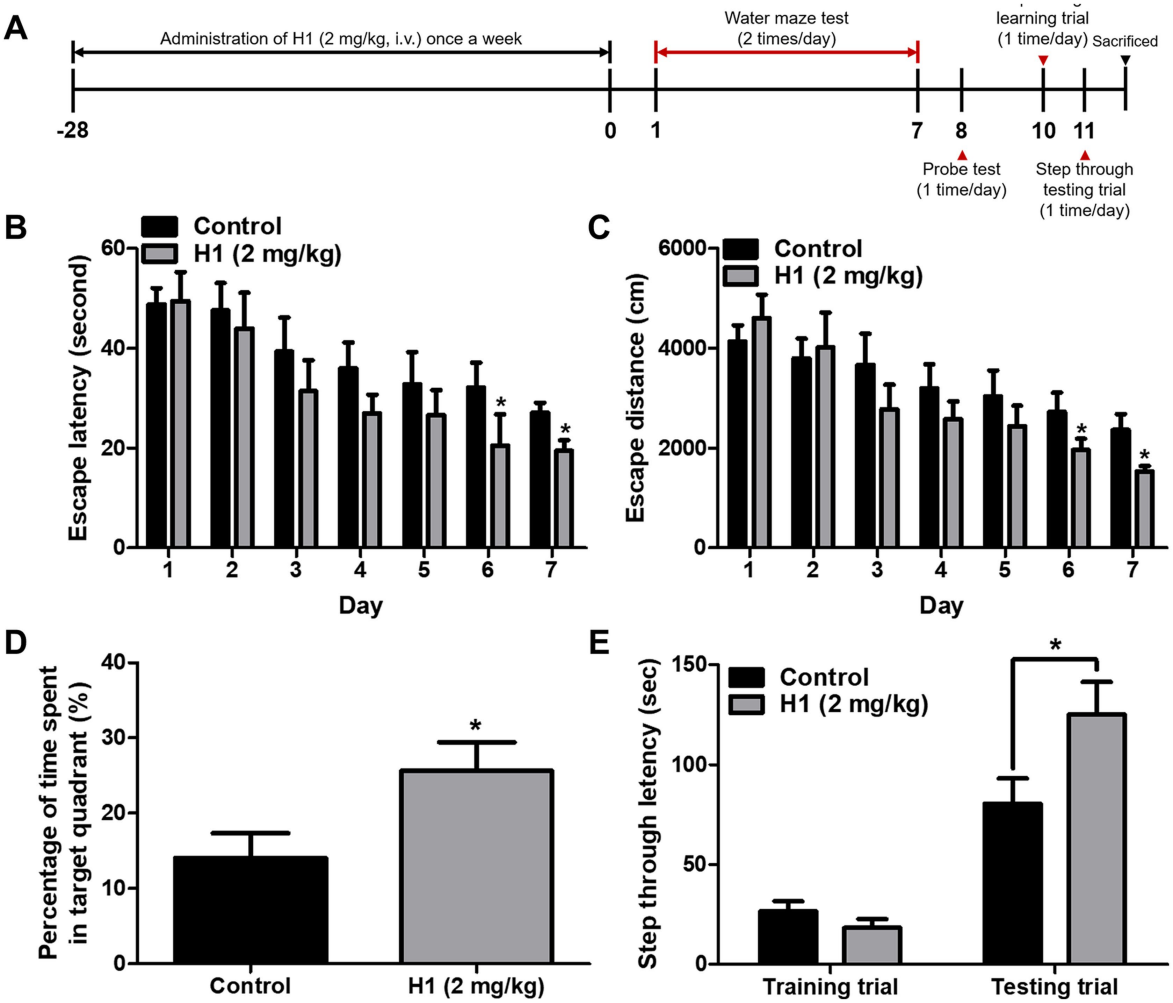
Chitinase-3-like 1 (CHI3L1) mAb attenuated memory dysfunction. **(A)** A timeline have been described that demonstrate the administration of CHI3L1 mAb (H1) and the assessment of cognitive function in Tg2576 mice. To investigate effect of H1 on memory impairment, we carried out **(B,C)** the water maze test, **(D)** the probe test, and **(E)** the step-through type passive avoidance test. Memory and learning ability in Tg2576 were determined by the escape latencies (**B**, sec) and escape distance (**C**, cm) for 6 days, and time spent in target quadrant (D, %) in the probe test.

To assess the effect of H1 antibody on the memory impairment in Tg2576 AD mouse model, H1 (2 mg/kg) was intravenously injected to Tg2576 mice once a week for 1 month. After 4 weeks of administration, a series of behavioral tests were conducted to evaluate learning ability and memory of the Tg2576 mice (Figure 1A). The spatial learning and memory abilities in Tg2576 mice were assessed by the water maze test. On the final day of the water maze, the mean escape latency and swimming distance of the control group were about 27.07 ± 2.0 s and 2,362 ± 319.1 cm, respectively. The H1-treated group showed significantly decreased the mean escape latency and swimming distance compared to that of the control group, which were 19.49 ± 2.1 s (0.72-fold shorter) and 1,530 ± 110.2 cm (0.65-fold shorter), respectively (*n* = 8–10, *p* = 0.0230 and *p* = 0.0369, respectively) (Figures 1B,C). In order to evaluate the effect of H1 therapy on memory consolidation in Tg2576 mice, the probe test was performed after removing the hidden platform in water the day after the final day of water maze testing. In the probe test, memory consolidation was determined by the percentage of the mean time spent in the target quadrant where the platform was located. The mean time spent in the target quadrant was significantly increased in the H1-treated group (25.65 ± 3.8%, 1.85-fold higher) compared to that in the control group (14.09 ± 3.3%) (*n* = 8–10, *p* = 0.0354) (Figure 1D). To investigate the effect of H1 on the memory retention ability of Tg2576 mice, the passive avoidance test was carried out. There was no significant difference between the two groups in the training trial, but H1-treated group showed higher an average step through latency (125.2 ± 16.2 s, 1.55-fold longer) than that in the control group (80.58 ± 12.6 s) in the testing trial (*n* = 8–10, *p* = 0.0447) (Figure 1E).

### 3.2 CHI3L1 mAb inhibits amyloidogenesis in Tg2576 mouse brain

**Figure 2:**
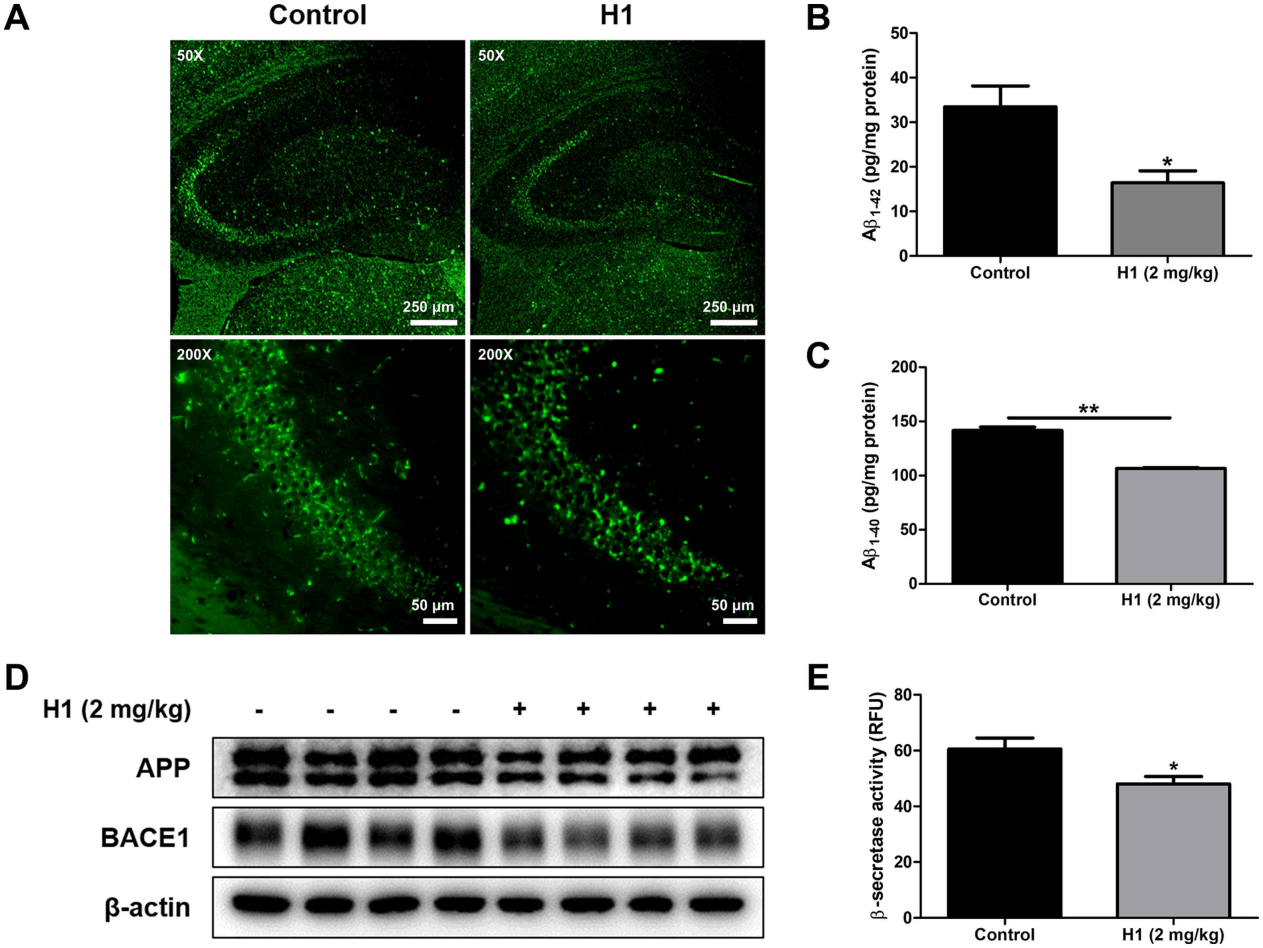
Inhibitory effects of Chitinase-3-like 1 (CHI3L1) mAb on amyloidogenesis. **(A)** The accumulation of amyloid beta (Aβ) plaques in hippocampus was determined by Thioflavin S staining. The scale bar for the low-magnification images (×50 objective lens) is 250 μm, and the scale bar for the high-magnification images (×200 objective lens) is 50 μm. **(B)** The levels of Aβ_1-42_ and **(C)** Aβ_1-40_ in Tg2576 mice brain were assessed using the specific enzyme-linked immunosorbent assay (ELISA) kits. **(D)** The expression of amyloid precursor protein (APP) and beta-secretase 1 (BACE1) were detected by Western blot. **(E)** The activity of β-secretase in mice brain was investigated by β-secretase activity assay kit.

The amyloid cascade hypothesis is by far the most well-known and accepted hypothesis of the causes of AD. According to this hypothesis, Αβ accumulation is highly associated with and is a major cause of AD. To investigate the effect of H1 on the Aβ plaque accumulation in the brain of Tg2576 mice, Thioflavin S staining was performed to stain β-sheet-rich structures of Aβ. The accumulation of Aβ plaques were reduced in the H1-treated group compared to that in the control group (Figure 2A). ELISA was performed to quantitatively measure the inhibitory effect of H1 on Aβ accumulation in the brain of Tg2576 mice. The Aβ_1-42_ level in the mouse hippocampus was 33.44 ± 4.7 pg./mg of protein in the control group and 16.41 ± 2.7 pg./mg of protein (0.49-fold lower) in H1-treated group (*n* = 8–10, *p* = 0.0144; Figure 2B). The Aβ_1-40_ level in the mouse hippocampus was 141.5 ± 3.3 pg./mg of protein in the control group and 106.5 ± 0.9 pg./mg of protein (0.75-fold lower) in H1-treated group (*n* = 8–10, *p <* 0.0001; Figure 2C). Taken together, the H1-treated group exhibits significantly lower Aβ levels than that of the control group. To determine how H1 inhibits Aβ accumulation, we measured the levels of proteins and the activity of β-secretase involving in Aβ production. The administration of H1 reduced the levels of APP and BACE1 in the brain of Tg2576 mice detected by the Western blot (Figure 2D), and significantly 0.79-fold reduced the β-secretase activity in the brain of Tg2576 mice (*n* = 8–10, *p* = 0.0350; Figure 2E).

### 3.3 Effect of CHI3L1 mAb against neuroinflammation in Tg2576 mouse brain and in murine microglial BV-2 cells

**Figure 3:**
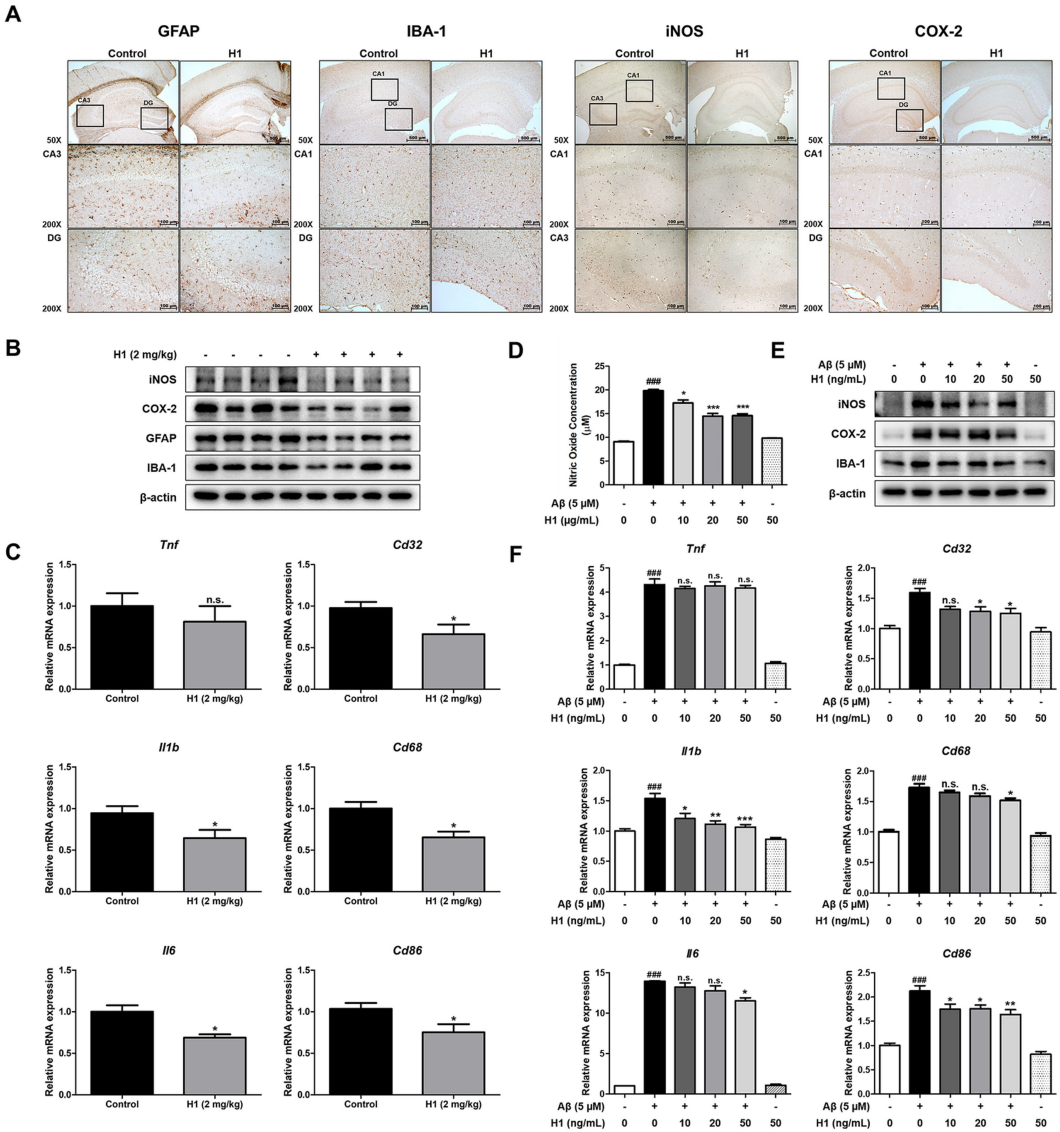
Effect of chitinase-3-like 1 (CHI3L1) mAb on neuroinflammation in Tg2576 mice brain and in BV-2 cells. **(A)** Expression of glial fibrillary acidic protein (GFAP), ionized calcium binding adaptor molecule 1 (IBA-1), inducible NO synthase (iNOS), and cyclooxygenase 2 (COX-2) in Tg2576 mice hippocampus were determined by immunohistochemistry analysis. The scale bar for the low-magnification images (×50 objective lens) is 500 μm, and the scale bar for the high-magnification images (×200 objective lens) is 100 μm. **(B)** Expression of iNOS, COX-2, GFAP, and IBA-1 in Tg2576 mice hippocampus were detected by Western blot. **(C)** The mRNA expression level of pro-inflammatory cytokines (*Tnf*, *Il1b*, and *Il6*) and M1 microglia phenotype markers (*Cd32, Cd68*, and *Cd86*) in Tg2576 mice hippocampus were assessed by qRT-PCR. BV-2 cells were treated with Aβ (5 μM) and H1 (10, 20, and 50 ng/mL) for 24 h. **(D)** The inhibitory effect of H1 on nitric oxide production in BV-2 cells were determined using nitric oxide assay kit in BV-2 cells. **(E)** Expression of iNOS, COX-2, and IBA-1 in BV-2 cells were detected by Western blot. **(F)** The mRNA expression level of pro-inflammatory cytokines (*Tnf*, *Il1b*, and *Il6*) and M1 microglia phenotype marker (*Cd32, Cd68*, and *Cd86*) in BV-2 cells were assessed by qRT-PCR.

Increasing evidence suggests that the development of AD is accompanied by neuroinflammation, such as the activation of astrocytes or microglia. In order to investigate the effect of H1 antibody on neuroinflammation, immunohistochemistry, Western blot, and qRT-PCR were performed to determine changes in factors associated with neuroinflammation between the two groups. The number of GFAP (the marker of reactive astrocyte)-reactive cells and IBA-1 (the marker of activated microglia)-reactive cells was reduced in the H1-treated group compared to that of the control group. The number of iNOS and COX-2-reactive cells involved in the neuroinflammatory response was reduced in the brain of H1-treated group (Figure 3A). Consistent with immunohistochemistry results, the expression of GFAP, IBA-1, iNOS, and COX-2 in Western blot results also significantly decreased in the brain of mouse treated with H1 (Figure 3B).

Increased production of inflammatory cytokines and differentiation into M1 microglia are well known indicators of neuroinflammation. qRT-PCR was performed to determine the levels of inflammatory cytokine production and the level of surface receptors as M1 microglia marker in mouse brain. The mRNA levels of pro-inflammatory cytokines such as *Tnf*, *Il1b*, and *Il6* in the brains of the H1-treated group were reduced compared to those in the control group except *Tnf* (*n* = 8–10; *Tnf*: *p* = 0.4593, 0.81-fold lower; *Il1b*: *p* = 0.0459, 0.68-fold lower; *Il6*: *p* = 0.0177, 0.69-fold lower) (Figure 3C). The mRNA levels of M1 microglia markers such as *Cd32*, *Cd68*, and *Cd86* in the brains of the H1-treated group were reduced compared to those in the control group (*n* = 8–10; *Cd32*: *p* = 0.0378, 0.68-fold lower; *Cd68*: *p* = 0.0167, 0.65-fold lower; *Cd86*: *p* = 0.0395, 0.75-fold lower) (Figure 3C). Activation of microglia is considered one of the major factors involved in neuroinflammation in AD. In order to explain the inhibitory effect of H1 antibody, nitric oxide (NO) concentration and expression levels of inflammatory proteins and cytokines in BV-2 cells were measured. The NO concentration was elevated in the Aβ-treated group, and the NO concentration was decreased by the treatment of H1 in a concentration-dependent manner (*n* = 4; *F*(5, 18) = 101.7, *p* < 0.0001) (Figure 3D). The expression of iNOS, COX-2, and IBA-1 was also significantly increased by Aβ, and decreased in the H1-treated groups (Figure 3E). Levels of pro-inflammatory cytokines, *Tnf*, *Il1b*, and *Il6*, were significantly increased by Aβ and decreased in a concentration-dependent manner in H1-treated groups except *Tnf* (*n* = 6–8; *Tnf*: *F*(5, 39) = 206.3, *p* < 0.0001; *Il1b*: *F*(5, 37) = 16.36, *p* < 0.0001; *Il6*: F(5, 39) = 228.5, *p* < 0.0001) (Figure 3F). Levels of M1 microglia markers, *Cd32*, *Cd68*, and *Cd86*, were significantly increased by Aβ and decreased in a concentration-dependent manner in H1-treated groups (*n* = 6–8; *Cd32*: *F*(5, 41) = 11.74, *p* < 0.0001; *Cd68*: *F*(5, 40) = 60.54, *p* < 0.0001; *Cd86*: F(5, 39) = 31.76, *p* < 0.0001) (Figure 3F).

### 3.4 Inhibitory effect of CHI3L1 mAb on ERK and NF-κB signaling pathway

**Figure 4:**
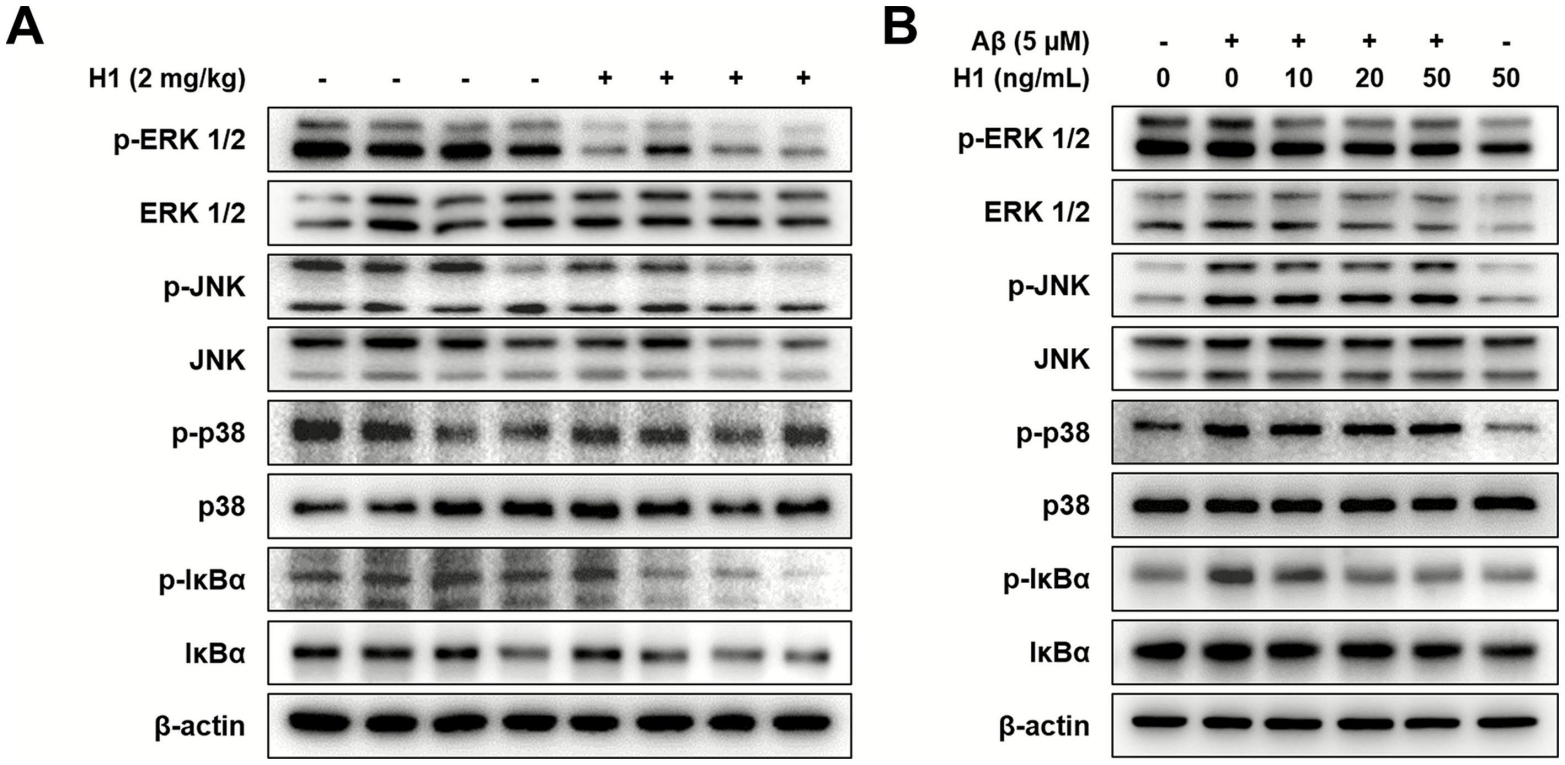
Effect of chitinase-3-like 1 (CHI3L1) mAb on ERK and NF-κB signaling pathway. **(A)** Level of p-extracellular signal-regulated kinases (ERK 1/2), ERK 1/2, p-c-jun N-terminal kinase (JNK), JNK, p-p38 mitogen-activated protein kinase (p38), p38, p-nuclear factor-kappa B inhibitor (IκBα), and IκBα were detected by Western blot in the Tg2576 mice brain. BV-2 cells were treated with Aβ (5 μM) and H1 (10, 20, and 50 ng/mL) for 24 h. **(B)** Level of p-ERK 1/2, ERK 1/2, p-JNK, JNK, p-p38, p38, p-IκBα, and IκBα were detected by Western blot.

Activation of MAPK or NF-κB signaling pathways is thought to contribute to AD pathogenesis through various mechanisms, including induction of neuronal apoptosis and APP phosphorylation (Kim and Choi, 2010; Sun et al., 2022). To investigate which signaling pathways the H1 antibody exerts its anti-inflammatory effects, we screened factors involved in MAPK and NF-κB signaling in Tg2576 mouse brain and BV-2 microglial cells using Western blot analysis. Among the factors involved in these signaling pathways, those that were decreased in the brains of the H1-treated group were p-ERK1/2 and p-IκBα (Figure 4A). In BV-2 cells, we also confirmed that phosphorylated ERK1/2 and IκBα induced by Aβ decreased as the H1 treatment concentration increased (Figure 4B). These results were consistent with those of our previous study using CHI3L1 inhibitory chemicals, K284-6111, suggesting that CHI3L1 inhibition reduces the activation of ERK and NF-κB signaling (Ham et al., 2020b).

### 3.5 Inhibitory effect of CHI3L1 mAb on neuroinflammatory responses induced by CHI3L1

**Figure 5:**
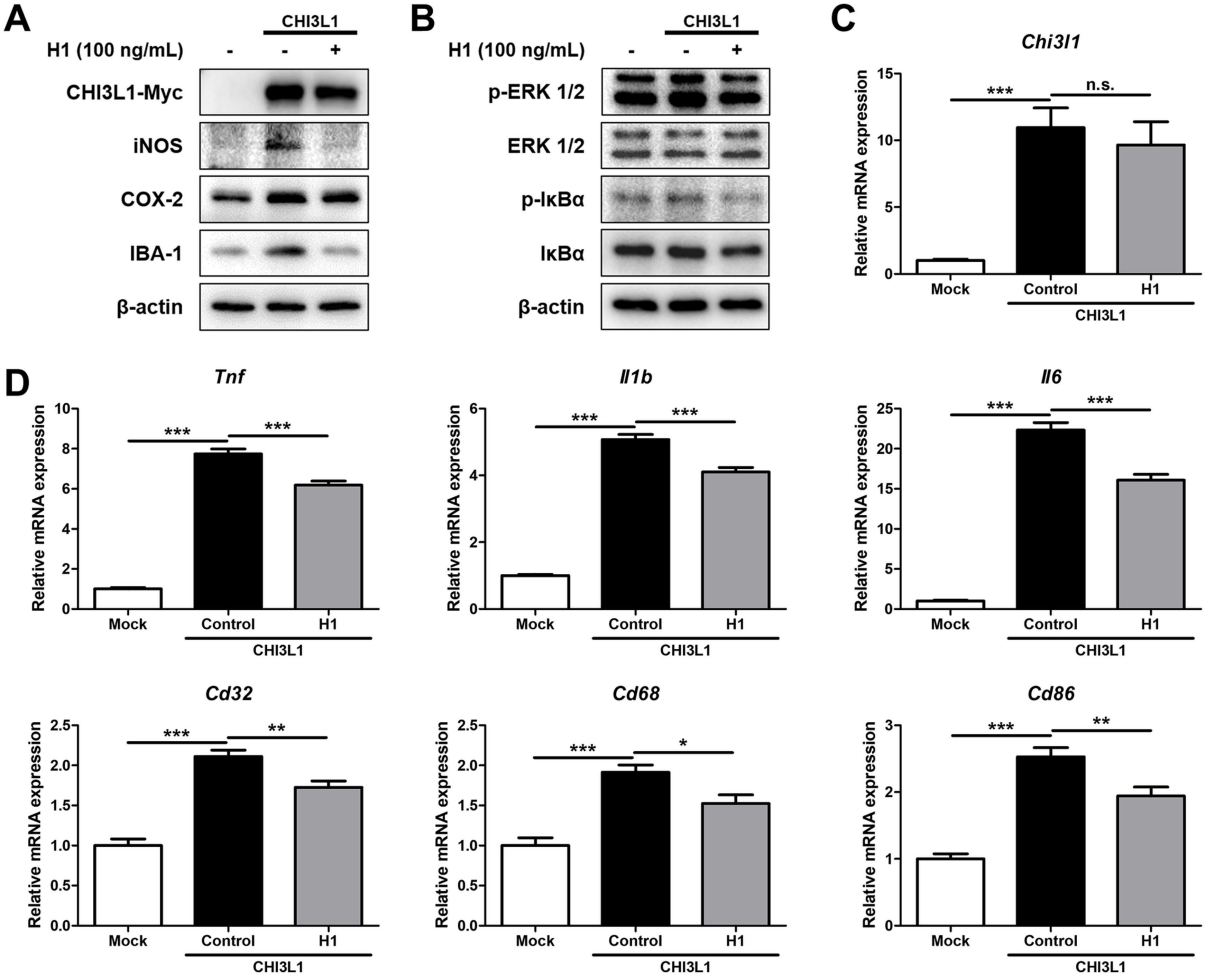
Effect of chitinase-3-like 1 (CHI3L1) mAb on neuroinflammation induced by CHI3L1. BV-2 cells were transfected with CHI3L1 plasmid vector. After 24 h, cells were treated with H1 (100 ng/mL) for 24 h. **(A)** Expression of inducible NO synthase (iNOS), cyclooxygenase 2 (COX-2), and ionized calcium binding adaptor molecule 1 (IBA-1) were detected by Western blot analysis using specific antibodies in BV-2 cells. **(B)** Level of p- extracellular signal-regulated kinases (ERK 1/2), ERK 1/2, p- nuclear factor-kappa B inhibitor (IκBα), and IκBα were detected by Western blot. (C, D, E) The mRNA expression level of CHI3L1, pro-inflammatory cytokines (*Tnf*, *Il1b*, and *Il6*), and M1 microglia phenotype marker (*Cd32, Cd68,* and *Cd86*) in BV-2 cells were assessed by quantitative real-time PCR (qRT-PCR).

To determine whether the H1 antibody exerts its anti-neuroinflammatory effect by targeting and inhibiting CHI3L1, BV-2 cells were transfected with the CHI3L1 expression vector and then treated with H1 (100 ng/mL) for 24 h. Western blot was used to compare inflammatory protein levels, and qRT-PCR was used to compare the expression levels of inflammatory cytokines and M1 surface markers between these groups. BV-2 cells transfected with CHI3L1 expression vector showed higher levels of iNOS, COX-2, and IBA-1, and higher phosphorylation levels of ERK and IκBα compared to those of cells transfected with the control vector. H1 treatment reduced the levels of these factors increased by CHI3L1 overexpression (Figures 5A, 5B). BV-2 cells transfected with CHI3L1 expression vector showed higher mRNA levels of pro-inflammatory cytokines, *Tnf*, *Il1b*, and *Il6*, and higher mRNA levels of M1 microglia surface markers, *Cd32*, *Cd68*, and *Cd86,* compared to those of cells transfected with the control vector. These expression levels were also significantly reduced in the H1 treatment group (*n* = 8; *Tnf*: F(2, 21) = 336.4, *p* < 0.0001; *Il1b*: F(2, 21) = 309.3, *p* < 0.0001; *Il6*: F(2, 21) = 258.0, *p* < 0.0001; *Cd32*: F(2, 21) = 48.24, *p* < 0.0001; *Cd68*: F(2, 21) = 20.90, *p* < 0.0001; *Cd86*: F(2, 21) = 41.56, *p* = 0.0001) (Figures 5D,E). In contrast, CHI3L1 increased by the transfection with the CHI3L1 expression vector was not decreased by H1 treatment (*n* = 6–8; *Chi3l1*: F(2, 21) = 16.79, *p* < 0.0001) (Figure 5C).

### 3.6 CHI3L1 mAb inhibits CHI3L1-induced HAX1 expression

**Figure 6:**
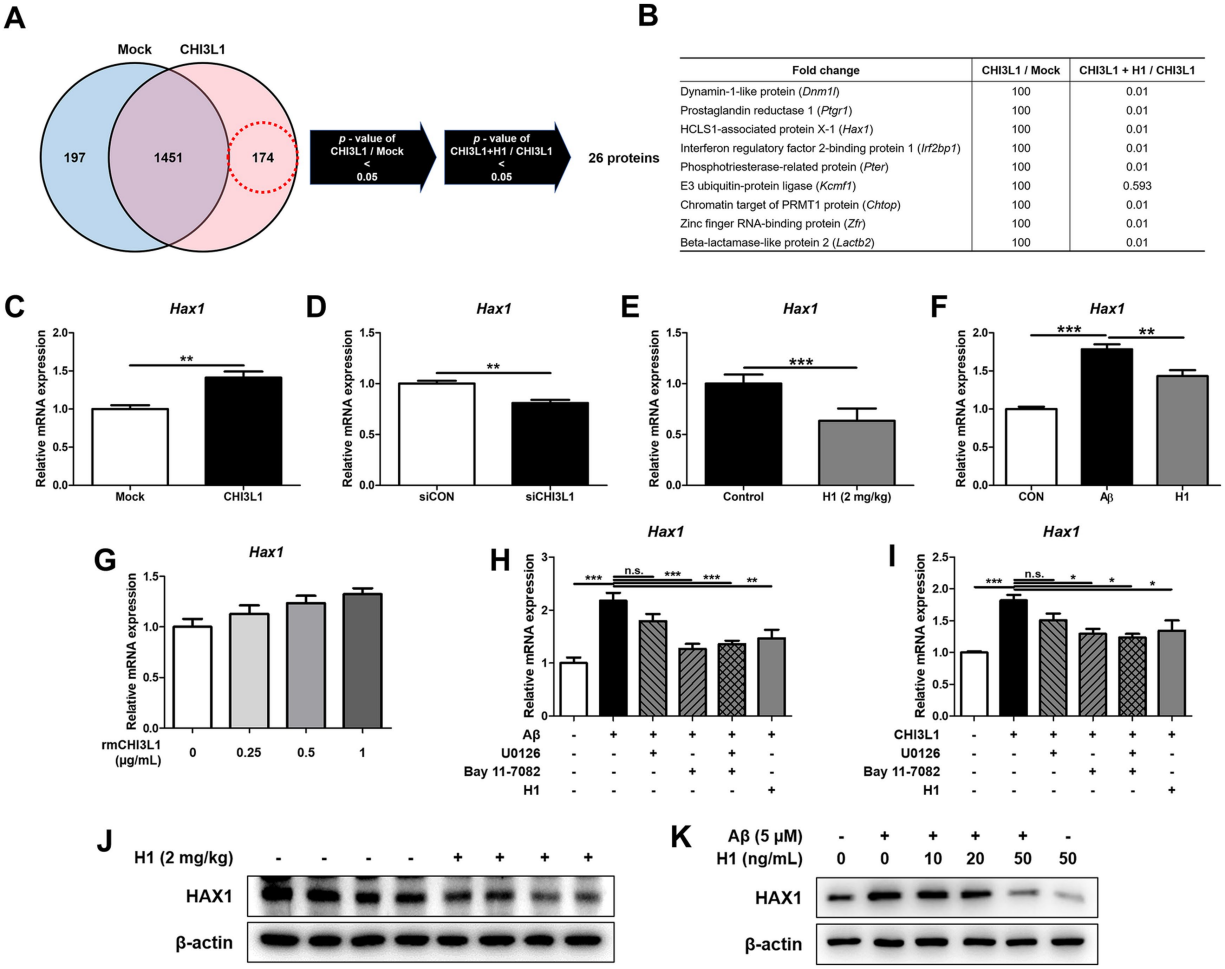
Chitinase-3-like 1 (CHI3L1) is associated with HCLS1-associated protein X-1 (HAX1). BV-2 cells were transfected with CHI3L1 plasmid vector. After 24 h, cells were treated with H1 (100 ng/mL) for 24 h. **(A)** Proteomic analysis using LC–MS/MS in BV-2 cells overexpressing CHI3L1. **(B)** List of selected nine proteins among the 26 candidates. **(C)** The mRNA expression level of *Hax1* in BV-2 cells overexpressing CHI3L1 were assessed by quantitative real-time PCR (qRT-PCR). BV-2 cells were transfected with CHI3L1 siRNA (20 nM). **(D)** After 24 h, the mRNA expression level of *Hax1* in BV-2 cells knockdowned CHI3L1 were assessed by qRT-PCR. **(E)** The mRNA expression level of *Haxl* in the Tg2576 mice brain. BV-2 cells were treated with Aβ (5 μM) and H1 (100 ng/mL) for 24 h. **(F)** The mRNA expression level of *Hax1* in BV-2 cells were assessed by qRT-PCR. BV-2 cells were treated with rmCHI3L1 (0.25, 0.5, and 1 μg/mL) for 24 h. **(G)** The mRNA expression level of *Hax1* in BV-2 cells were assessed by qRT-PCR. BV-2 cells were treated with Aβ (5 μM), H1 (100 ng/mL), U0126 (20 μM), and Bay 11–7,082 (5 μM) for 24 h. **(H)** The mRNA expression level of *Hax1* were assessed by qRT-PCR. BV-2 cells were treated with rmCHI3L1 (1 μg/mL), H1 (100 ng/mL), U0126 (20 μM), and Bay 11–7,082 (5 μM) for 24 h. **(I)** The mRNA expression level of *Hax1* were assessed by qRT-PCR. Expression of HAX1 were detected by Western blot **(J)** in the Tg2576 mice brain and **(K)** in the BV-2 cells.

To understand the anti-neuroinflammatory mechanism of CHI3L1 mAb, H1, we performed global proteomics analysis using mass spectrometry to quantify the proteins actually expressed in BV-2 cells transfected with CHI3L1 expression vector and then treated with H1 (100 ng/mL) for 24 h. We identified 26 proteins that were significantly increased in the CHI3L1 overexpressing group compared to the control group and that were significantly decreased by CHI3L1 inhibition through H1 treatment (Figure 6A). Among the 26 proteins, we selected 9 that were reported to be associated with inflammatory responses (Figure 6B) and analyzed their gene expression levels in CHI3L1 knockdowned BV-2 cells using qRT-PCR. The deficiency of CHI3L1 using small interfering RNA (siRNA) significantly decreased mRNA levels of only four proteins: *Dnm1l*, *Ptgr1*, *Hax1*, and *Pter* (*n* = 6; *Dnm1l*: *p* = 0.0153; *Ptgr1*: *p* = 0.0077; *Hax1*: *p* = 0.0011; *Irf2bp1*: *p* = 0.6753; *Pter*: *p* = 0.0251; *Kcmf1*: *p* = 0.9679; *Chtop*: *p* = 0.6288; *Zfr*: *p* = 0.6245; *Lactb2*: *p* = 0.4698) (Figure 6D, Supplementary Figure S2). The expression levels of these four proteins, whose expression was significantly reduced by CHI3L1 knockdown, were analyzed by qRT-PCR in the brains of Tg2576 mice treated with H1. The mRNA levels of *Hax1* and *Pter* in the brains of the H1-treated group were significantly reduced compared to those in the control group (*n* = 8–10; *Dnm1l*: *p* = 0.2649; *Ptgr1*: *p* = 0.4558; *Hax1*: *p* = 0.0380; *Pter*: *p* = 0.0007) (Figure 6E, Supplementary Figure S3). The expression levels of these *Hax1* and *Pter* were analyzed by qRT-PCR in Aβ-induced BV-2 cells treated with H1. The mRNA level of *Hax1* was significantly increased by Aβ and significantly decreased by H1 treatment (*n* = 7–8; *Hax1*: *F*(2, 20) = 36.82, *p* < 0.0001; *Pter*: F(2, 20) = 1.226, *p* = 0.3145) (Figure 6F, Supplementary Figure S4). To determine whether HAX1 expression is regulated by exogenously secreted CHI3L1, BV-2 cells were treated with rmCHI3L1 and mRNA levels of *Hax1* were measured by qRT-PCR. The mRNA levels of *Hax1* were increased in a concentration-dependent manner by rmCHI3L1 treatment in BV-2 cells (*n* = 7–8; *Hax1*: *F*(3, 20) = 3.517, *p* = 0.0340) (Figure 6G). To determine the effect of H1 on HAX1 levels, Western blot analysis was carried out in the brain of Tg2576 mice and Aβ-induced BV-2 cells. The levels of HAX1 in the brain of Tg2576 mice were lower by H1 treatment (Figure 6J), and the levels of HAX1 in Aβ-induced BV-2 cells were decreased in a concentration-dependent manner by H1 treatment (Figure 6K).

To explore the pathway by which HAX1 is regulated, BV-2 cells in which HAX1 is upregulated by Aβ treatment were treated with ERK1/2 inhibitor (U0126) or NF-κB inhibitor (Bay 11–7,082), and *Hax1* expression was measured by qRT-PCR (Figure 6H). The increased *Hax1* level was decreased by U0126 treatment or Bay 11–7,082 treatment, but was decreased more by Bay 11–7,082 treatment, and even when U0126 and Bay 11–7,082 were treated together, the *Hax1* expression level was similar to when Bay 11–7,082 was treated alone. These results were also observed when ERK or NF-κB inhibitors were treated with BV-2 cells in which HAX1 expression was increased due to rmCHI3L1 treatment (Figure 6I).

## 4 Discussion

Our previous studies showed that the CHI3L1 targeting compound, K284-6111 and G721-0282, could alleviate neuroinflammation by inhibiting CHI3L1 (Ham et al., 2020a; Ham et al., 2020b). In this study, we found that monoclonal antibody (mAb) therapy targeting CHI3L1 has improved effects on memory impairment and cognitive function in Tg2576 AD mouse model. In the previous study, we screened the high-affinity anti-CHI3L1 mAb in a human synthetic Fab phage display library, of which clone H1, which showed the highest ability to inhibit the metastasis and tumor growth *in vivo,* was selected (Kang et al., 2020). Consistent with the memory and cognitive palliative effects of previous CHI3L1 targeting compounds, H1 therapy was effective in inhibiting amyloidogenesis and neuroinflammation in Tg2576 mice. Moreover, H1 therapy inhibited the activation of NF-κB signaling pathways involved in neuroinflammatory responses associated with AD development and progression.

The blood–brain barrier (BBB) consists of a tightly connected endothelial layer within the cerebral microvasculature that serves to shield neural tissue from circulating peripheral elements, thereby preserving the specialized environment of the central nervous system (CNS) (Wu et al., 2023). Due to this selective barrier function, large biomolecules such as antibodies are generally unable to penetrate the CNS under normal physiological conditions. However, multiple studies have demonstrated that in patients with AD, BBB integrity becomes compromised at early disease stages—preceding even hippocampal atrophy—resulting in increased permeability compared to cognitively healthy individuals (Sweeney et al., 2018). This altered BBB permeability has clinical relevance; the U.S. Food and Drug Administration (FDA) has granted approval to several anti-amyloid monoclonal antibodies—including aducanumab (Aduhelm^®^), lecanemab (Leqembi^®^), and donanemab (Kisunla™)—for their ability to reduce amyloid plaque burden in the brains of individuals with Alzheimer’s disease (Kim et al., 2025). To evaluate whether H1 is capable of crossing the blood–brain barrier and exerting its effects within the brain, we assessed the distribution of H1 conjugated with ICG in both Tg2576 mice and an LPS-induced AD-like mouse model. Although the overall permeability of H1 across the BBB was not markedly high, our findings indicated that H1 was able to partially penetrate the barrier in these AD model mice. This observation suggests that H1 may reach brain and modulate CHI3L1 levels, thereby exerting potential therapeutic effects in the context of Alzheimer’s pathology. In this study, we could not perform quantitative analyses of BBB penetration, such as brain/plasma fluorescence ratios or biodistribution measurements. Future studies incorporating these analyses could provide more precise information on H1’s central penetration and kinetics, aiding tshe optimization of dosing strategies for potential clinical applications.

Beyond the classical hallmarks of amyloid-β deposition and tau pathology, sustained neuroinflammation has gained increasing recognition as a core pathological feature of AD (Heppner et al., 2015; Harach et al., 2017). In particular, the abnormal activation of microglia and astrocytes plays a pivotal role in mediating neuronal damage through the enhanced production of pro-inflammatory cytokines, inducible nitric oxide, and prostaglandin-associated inflammatory mediators (DiSabato et al., 2016; Su et al., 2016). Clinical studies have consistently demonstrated the activation of microglia and astrocytes in Alzheimer’s disease (Serrano-Pozo et al., 2011; Pereira et al., 2021). Pereira et al. reported a significant association between plasma levels of GFAP and elevated aβ-PET signal across all amyloid-positive individuals, indicating astrocytic reactivity in relation to amyloid burden (Pereira et al., 2021). Similarly, Serrano-Pozo et al. observed a progressive increase in glial activation throughout the course of the disease in AD patients, with markedly activated microglia clustering around dense-core amyloid plaques (Serrano-Pozo et al., 2011). In serum of AD patients, pro-inflammatory cytokines such as TNF-*α*, IL-1β, and IL-6 have been reported to be higher than normal (Khemka et al., 2014; Domingues et al., 2017). Taipa et al. analyzed concentrations of 27 cytokines in cerebrospinal fluid and found that levels of TNF-α were elevated in AD patients compared to non-demented controls (Taipa et al., 2019). In a separate meta-analysis, Swardfager et al. reported that individuals with AD exhibited higher circulating levels of pro-inflammatory cytokines, including IL-6, TNF-α, and IL-1β, relative to healthy participants (Swardfager et al., 2010). Michelucci et al. reported that microglia exposed to oligomeric Aβ adopted a pro-inflammatory M1 phenotype of microglia that closely resembled the profile induced by LPS treatment, accompanied by impaired phagocytic activity, ultimately reducing Aβ clearance and contributing to AD progression (Kim et al., 2025). Similarly, Cui et al. demonstrated that M1 markers were upregulated in APP/PS1 transgenic mice compared to WT, and that this increase was also observed *in vitro* following Aβ stimulation, consistent with the *in vivo* findings (Cui et al., 2020). Administration of H1 in Tg2576 mice significantly attenuated the expression of inflammatory mediators, including IL-1β, IL-6, and M1 phenotype-associated surface markers, while concurrently suppressing the activation of microglia and astrocytes in the brain. Consistent effects were observed in BV-2 murine microglial cells, in which Aβ-induced upregulation of pro-inflammatory cytokines and M1 markers was mitigated by H1 treatment. Although the reduction in TNF-α expression did not reach statistical significance, a decreasing trend was evident, suggesting that an increased number of experimental replicates may reveal a statistically significant effect. Upregulation of iNOS and the associated increase in NO production, along with enhanced expression of COX-2, have been linked to the advancement of AD pathology (O'banion, 1999; Xiang et al., 2002; Medeiros et al., 2007). Elevated levels of both iNOS and COX-2 have been reported in postmortem brain tissue of AD patients (Lee et al., 1999; Wang et al., 2014). Accumulating evidence suggests that genetic deletion of iNOS can mitigate Aβ-mediated neurotoxicity, while COX-2 has been shown to modulate APP processing, thereby contributing to the acceleration of cerebral amyloid accumulation (O'banion, 1999; Xiang et al., 2002; Nathan et al., 2005). In this study, we found that treatment with H1 markedly suppressed the expression of both iNOS and COX-2 in *in vivo* and *in vitro* AD models. Collectively, these data indicate that H1 may exert therapeutic potential in AD by attenuating neuroinflammatory responses.

Elevated expression of CHI3L1 has been observed in a range of neurological disorders, including AD, amyotrophic lateral sclerosis, multiple sclerosis, and schizophrenia (Yang et al., 2008). Through integrative analysis using the Open Targets Platform and literature-based scoring, AD emerged as the neurological condition most strongly associated with the CHI3L1 gene (Muszynski et al., 2017; Yu et al., 2024). CHI3L1 has been proposed as a surrogate indicator of neuroinflammation and may possess prognostic relevance as a preclinical biomarker for AD (Muszynski et al., 2017). Elevated plasma levels of CHI3L1 in individuals with early-stage AD support its potential utility in early detection (Choi et al., 2011). In our previous work, serum CHI3L1 concentrations were significantly higher in AD patients compared to cognitively normal controls, and receiver operating characteristic (ROC) analysis indicated its promise as a reliable diagnostic indicator (Ham et al., 2024). Several studies have linked CHI3L1 upregulation to microglial activation, neuronal degeneration, and hippocampal injury (Roveta et al., 2024). Emerging evidence suggests that CHI3L1 contributes to amyloid plaque formation, as demonstrated by studies in APP/PS1 transgenic mice lacking CHI3L1, in which genetic deletion of CHI3L1 attenuated AD-like features, suppressed glial phagocytic responses, and reduces amyloid burden (Lananna et al., 2020). Neuroinflammatory responses including nurotoxic Aβ plaques in the brain activate microglia, which subsequently release CHI3L1 (Yeo et al., 2019). According to Sanfilippo et al. (2017) the CHI3L1 secreted by glial cells such as microglia and astrocytes may promote infiltration of peripheral immune cells, including macrophages and monocytes, into the brain, potentially exacerbating neuronal damage. Supporting this, Pranzatelli et al. (2017) observed elevated CHI3L1 concentrations in both cerebrospinal fluid and blood samples from pediatric patients with inflammatory neurological diseases. In our previous study, we modeled AD by administering Aβ directly into the brains of both CHI3L1 KO and WT mice (Ham et al., 2024). The CHI3L1 KO group exhibited markedly reduced expression levels of neuroinflammatory markers, including those associated with activated microglia and astrocytes, as well as lower concentrations of pro-inflammatory cytokines in the brain, compared to WT controls. Additionally, knockdown of CHI3L1 in Aβ-stimulated BV-2 microglial cells resulted in significant suppression of inflammation-associated mediators. Consistently, in the current study, enforced CHI3L1 expression in BV-2 cells enhanced production of inflammatory cytokines and increased markers indicative of M1 polarization. Although some previous reports suggest CHI3L1 may be linked to M2 polarization (Jingjing et al., 2017; Steenbrugge et al., 2018; Xu et al., 2019), our findings in H1-treated Tg2576 mice demonstrated a decline in transcripts associated with M1 activation—specifically *Il1b*, *Il6*, and other inflammatory mediators. Furthermore, CHI3L1 overexpression upregulated pro-inflammatory markers including *Tnf, Il1b, Il6, Cd32, Cd68,* and *Cd86,* all of which were significantly downregulated following H1 administration.

The NF-κB and ERK signaling pathways are well-recognized regulators of immune-related gene expression and play pivotal roles in neuroinflammatory cascades mediated by glial cells during the progression of neurodegenerative disorders, including AD disease, Parkinson’s disease, and amyotrophic lateral sclerosis (Chen et al., 2012; Albert-Gasco et al., 2020). Several studies have reported elevated NF-κB activity in the cerebral cortex of individuals with AD, which may not only amplify inflammatory responses but also enhance BACE1 expression, thereby accelerating Aβ plaque development (Chen et al., 2012; Kaltschmidt et al., 2024). Numerous studies have reported that ERK signaling is closely associated with neuroinflammation (Park et al., 2017; Liu et al., 2023; Mostafa and Asaad, 2023; Wang et al., 2024). Shengpeng Liu and colleagues demonstrated that Secukinumab attenuated neuroinflammation via the PKCβ/ERK/NF-κB signaling pathway, while Park et al. reported that Asiatic acid alleviated methamphetamine-induced neuroinflammation through the NF-κB/STAT3/ERK pathway (Park et al., 2017; Liu et al., 2023). Collectively, these findings suggest a strong correlation between ERK activation and neuroinflammatory processes. Additionally, phosphorylated ERK has been identified as an upstream regulator of several AD risk-associated genes, including *Bin1, Cd33, Trem2,* and *Cnn2* (Chen et al., 2021). A large-scale quantitative proteomic analysis of postmortem AD brains revealed that ERK signaling was the only MAPK pathway positively correlated with neuropathological severity (Chen et al., 2021; Almasoudi et al., 2025). In our study, treatment with H1 attenuated NF-κB and ERK signaling in both the brains of Tg2576 mice and Aβ-induced BV-2 cells, leading to a reduction in inflammation-related mediators. Furthermore, the activation of NF-κB and ERK pathways induced by CHI3L1 overexpression in BV-2 cells was also diminished following H1 administration.

HAX1 is an intracellular protein implicated in maintaining mitochondrial function and preventing programmed cell death, and has also been associated with hematopoietic cell survival and cytoskeletal dynamics (Chao et al., 2008; Trebinska-Stryjewska et al., 2023). Although it is not traditionally categorized as an inflammatory modulator, emerging evidence suggests its broader role in cellular stress adaptation, including within immune-responsive contexts (Fadeel and Grzybowska, 2009; Balcerak et al., 2019). In our study, overexpression or exogenous treatment of CHI3L1 led to a marked increase in HAX1 expression, whereas silencing CHI3L1 or blocking its activity with H1 significantly reduced HAX1 levels. These findings indicate that HAX1 expression is positively regulated by CHI3L1 in microglial cells. Although we examined changes in HAX1 expression following CHI3L1 knockdown or H1 treatment, and additionally assessed HAX1 regulation after applying ERK and NF-κB inhibitors, these results do not conclusively determine through which specific downstream signaling mechanism HAX1 may accelerate inflammatory responses. While HAX1 induction may represent a compensatory mechanism aimed at preserving cell viability and counteracting inflammation-induced pyroptosis or apoptosis, it is also possible that elevated HAX1 expression contributes to amplifying glial activation and sustaining inflammation, thereby exacerbating pathological processes in AD. Thus, further investigation is required to elucidate the mechanistic role of the CHI3L1–HAX1 axis as a context-dependent modulator of neuroinflammation.

Our study provides compelling evidence that targeting CHI3L1 with a monoclonal antibody (H1) mitigates key pathological features of AD in Tg2576 mouse, including neuroinflammation, amyloid deposition, and cognitive impairment. By inhibiting the ERK and NF-κB pathways and downregulating HAX1, H1 exerts broad anti-inflammatory and neuroprotective effects. Given the multifactorial nature of AD, therapeutic strategies directed at upstream regulators such as CHI3L1 may offer more comprehensive and fundamental advantages than conventional approaches focusing solely on Aβ. However, although this study indirectly indicated neuroprotective effects through reduced activation of glial cells and decreased inflammatory cytokine levels, we were unable to directly quantify neuronal cell death or elucidate the underlying mechanisms in detail, representing a limitation of this work. Future studies should investigate the direct effects of CHI3L1 inhibition on neuronal survival using multiple markers of neuronal death and further clarify the associated molecular pathways. In addition, our evaluation of H1 was primarily focused on its effects within the brain. While H1 was confirmed to cross the BBB in AD mouse models, it is likely that the majority of the antibody remains distributed in the periphery. Therefore, future research should explore the peripheral actions of H1, particularly its potential to modulate systemic and peripheral immune responses, which may further clarify both the role of CHI3L1 in AD pathogenesis and the therapeutic potential of H1. Further investigation is required to address potential translational challenges associated with therapeutic antibodies. In particular, the immunogenicity, long-term safety, and optimal delivery strategies for CHI3L1-targeting antibodies remain to be fully elucidated. Future studies should systematically evaluate immune responses following repeated administration, assess long-term toxicity profiles, and develop more efficient delivery approaches to enhance CNS penetration while minimizing peripheral accumulation.

## 5 Conclusion

In the present study, we demonstrate that targeting CHI3L1 with the monoclonal antibody (H1) alleviates key pathological features of Alzheimer’s disease, including neuroinflammation, amyloid accumulation, and cognitive impairment, in the Tg2576 mouse model. H1 treatment inhibited ERK and NF-κB signaling pathways and reduced the expression of pro-inflammatory cytokines. These results suggest that CHI3L1 plays a central role in AD-related neuroinflammation and may serve as a promising upstream therapeutic target. By addressing both inflammatory and amyloidogenic pathways, CHI3L1-targeted therapy offers a multifaceted approach beyond conventional Aβ-focused treatments.

## Data Availability

The original contributions presented in the study are included in the article/Supplementary material, further inquiries can be directed to the corresponding authors.
