# Supplementary material for: CHI3L1 monoclonal antibody therapy mitigates cognitive impairment by inhibiting neuroinflammation through ERK and NF-κB pathway in Tg2576 mice
Source: Front Mol Neurosci. 2026 Jan 7;18:1728279. doi: 10.3389/fnmol.2025.1728279 (PMC12819630; doi:10.3389/fnmol.2025.1728279)
Supplement: Supplementary file 1 [file Table_1.DOCX]

**Supplementary information for**

**CHI3L1 monoclonal antibody therapy mitigates cognitive impairment by inhibiting neuroinflammation through ERK and NF-κB pathway in Tg2576 mice**

Hyeon Joo Ham^1^, Seung Sik Park^1^, Yong Sun Lee^1^, Tae Hun Kim^1^, Dong Ju Son^1^, Ji-Hun Kim^1^, Key-Hwan Lim^1^, Hanseul Park^1^, Hye Jin Lee^1^, Jaesuk Yun^1^, Sang-Bae Han^1^, Min Ki Choi^1^, and Jin Tae Hong^1^

^1^College of Pharmacy and Medical Research Center, Chungbuk National University, 194-21, Osongsaengmyeong 1-ro, Cheongju, North Chungcheong, 28160, Republic of Korea

*Correspondence: Dr. Jin Tae Hong (jinthong@chungbuk.ac.kr), College of Pharmacy and Medical Research Center, Chungbuk National University, 194-21, Osongsaengmyeong 1-ro, Cheongju, North Chungcheong, 28160, Republic of Korea, Tel: +82-043-261-2813, Fax: +82-043-268-2732.

**Supplementary Table S1. List and sequences of qPCR primers for mRNA expression**

|  | **Forward (5’→3’)** | **Reverse (5’→3’)** | **Species** |
| --- | --- | --- | --- |
| ***β-actin*** | GGCTGTATTCCCCTCCATCG | CCAGTTGGTAACAATGCCATGT | Mouse |
| ***Tnf*** | TCTTCTCATTCCTGCTTGTGG | CACTTGGTGGTTTGCTACGA | Mouse |
| ***Il1b*** | CCTTCCAGGATGAGGACATGA | TGAGTCACAGAGGATGGGCTC | Mouse |
| ***Il6*** | GAGGATACCACTCCCAACAGACC | AAGTGCATCATCGTTGTTCATACA | Mouse |
| ***Cd32*** | AATCCTGCCGTTCCTACTGATC | GTGTCACCGTGTCTTCCTTGAG | Mouse |
| ***Cd68*** | TTCTGCTGTGGAAATGCAAG | AGAGGGGCTGGTAGGTTGAT | Mouse |
| ***Cd86*** | TGTTTCCGTGGAGACGCAAG | TTGAGCCTTTGTAAATGGGCA | Mouse |
| ***Chi3l1*** | GTACAAGCTGGTCTGCTACTTC | ATGTGCTAAGCATGTTGTCGC | Mouse |
| ***Hax1*** | TTCCCACTCTCCTGAACTTCC | GGTTGGTGACTATCTGGGTACT | Mouse |
| ***Dnm1l*** | TTACGGTTCCCTAAACTTCACG | GTCACGGGCAACCTTTTACGA | Mouse |
| ***Ptgr1*** | GAAGGCTTCCCTACGGACG | GGCTGCAACTCTCATGTAAGGA | Mouse |
| ***Irf2bp1*** | GGTGCGTGAATTTCGAGGG | CTAGGTCCTTGGAAGTCGGGT | Mouse |
| ***Pter*** | TACGATCTCTTTGGTACGGAACT | CTCATCCACTAGAAAATGGACCC | Mouse |
| ***Kcmf1*** | TCGAGGTCGCAGATATAAGTGT | GTGGTCAGTTGTATGCCTTGTT | Mouse |
| ***Chtop*** | TAGGGCGTGGAGCTATGGG | CCCTCTTCCCCGACCTATCA | Mouse |
| ***Zfr*** | GCGACCGGCAACTACTTTG | GATGGGAATAGGCTACACCCG | Mouse |
| ***Lactb2*** | GGTCCCATGACCCTGCAAG | GGATTGCTGTGTCAAACTCAAC | Mouse |

**Supplementary figure S1. The analysis of biodistribution of H1 antibody using *ex vivo* imaging**

**
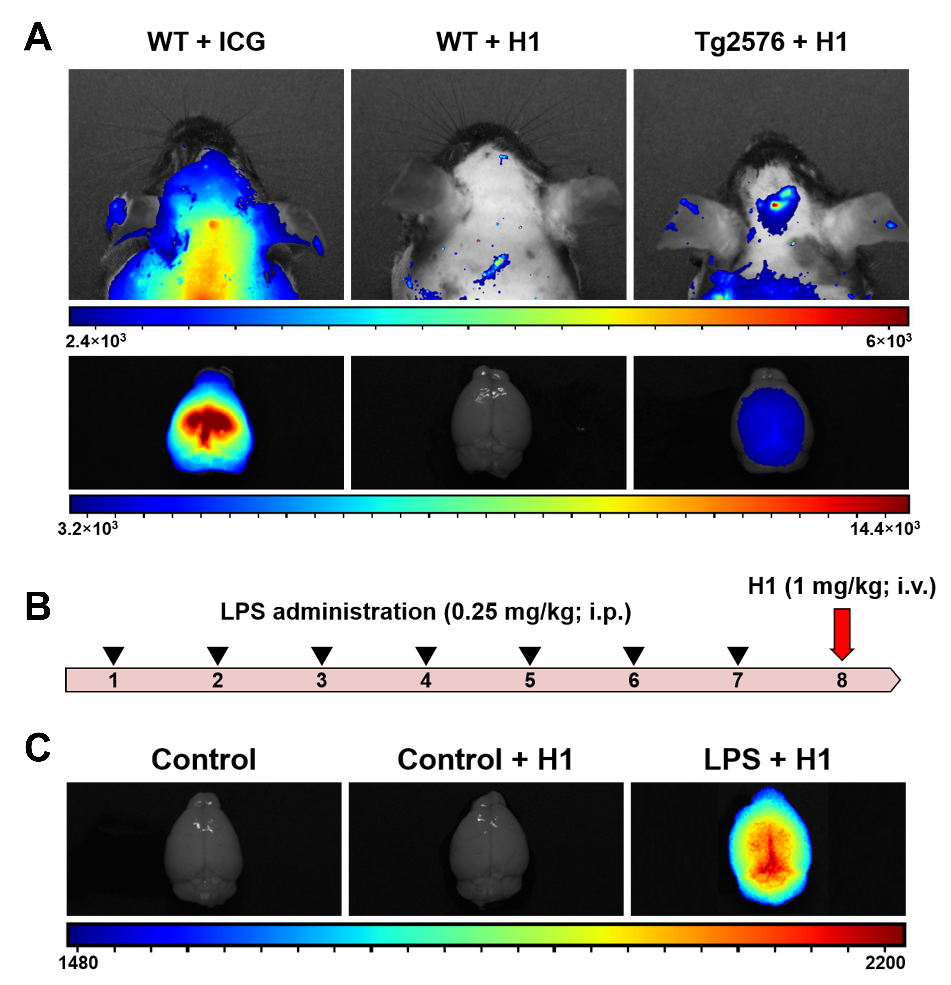
**

(A) Fluorescence image of mouse brain to assess penetration of H1-ICG conjugate in Tg2576 AD mouse and WT mouse. (B) A timeline demonstrating the LPS administration in WT mice. (C) Fluorescence image of mouse brain to assess penetration of H1-ICG conjugate in LPS induced AD-like mouse and control mouse.

**Supplementary figure S2. Relative mRNA expression levels in CHI3L1 knockdowned BV-2 cells**

**
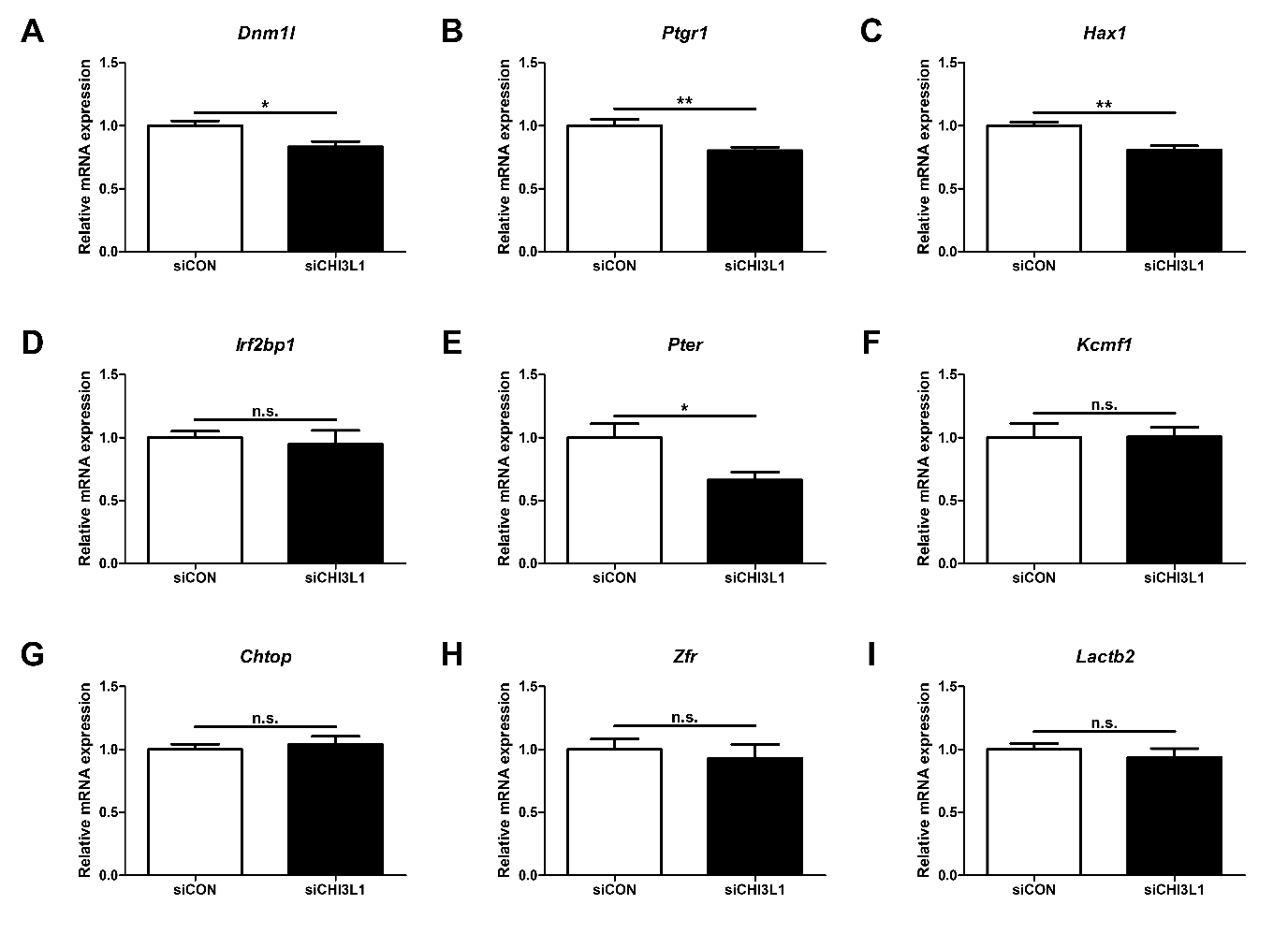
**

BV-2 cells were transfected with CHI3L1 siRNA (20 nM). The mRNA expression level of (A) *Dnm1l,* (B) *Ptgr1,* (C) *Hax1,* (D) *Irf2bp1,* (E) *Pter,* (F) *Kcmf1,* (G) *Chtop,* (H) *Zfr,* and (I) *Lactb2* in BV-2 cells were assessed by qRT-PCR. Each value is mean ± S.E.M. from 6 samples. *, Significantly different be-tween the two groups (p < 0.05); **, Significantly different between the two groups (p < 0.01).

**Supplementary figure S3. Relative mRNA expression levels in Tg2576 mice brain**

**
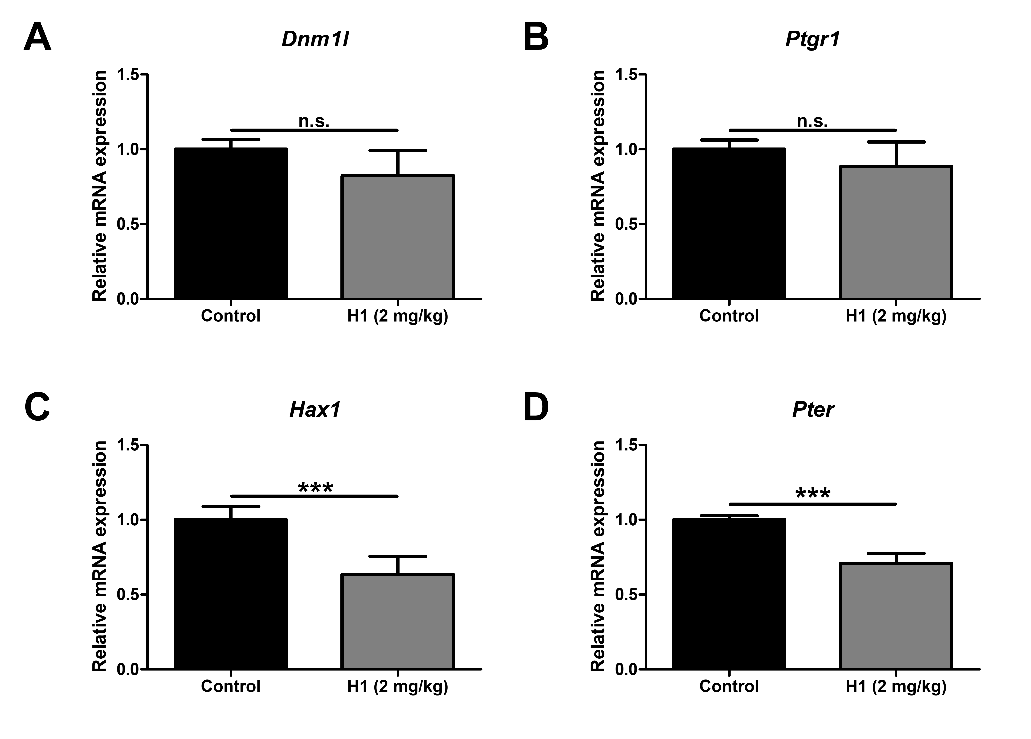
**

The mRNA expression level of (A) *Dnm1l,* (B) *Ptgr1,* (C) *Hax1,* and (D) *Pter* in Tg2576 mice hippocampus were assessed by qRT-PCR. Each value is mean ± S.E.M. from 6 samples. *, Significantly different be-tween the two groups (p < 0.05); **, Significantly different between the two groups (p < 0.01).

**Supplementary figure S4. Relative mRNA expression levels in Aβ-induced BV-2 cells**

**
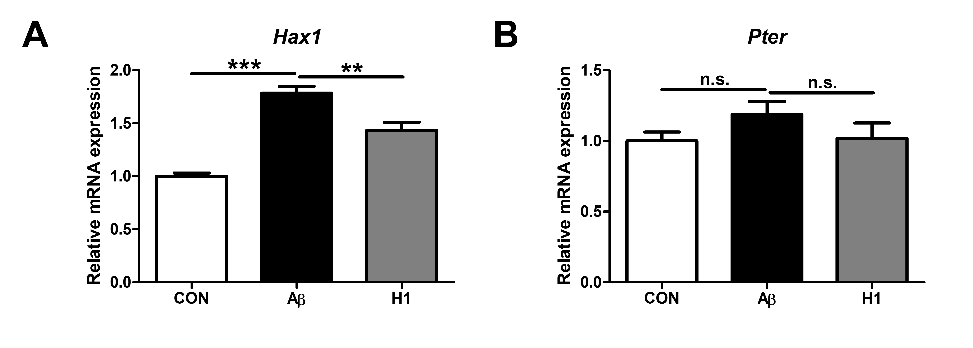
**

BV-2 cells were treated with Aβ (5 µM) and H1 (50 µg/mL). The mRNA expression level of (A) *Hax1* and (B) *Ptx3* in BV-2 cells were assessed by qRT-PCR. Each value is mean ± S.E.M. from 7-8 samples. **, Significantly different between the two groups (p < 0.01); ***, Significantly different between the two groups (p < 0.001).
